# The association of paid medical and caregiving leave with the economic security and wellbeing of service sector workers

**DOI:** 10.1186/s12889-021-11999-9

**Published:** 2021-11-01

**Authors:** Julia M. Goodman, Daniel Schneider

**Affiliations:** 1 Oregon Health & Science University, Portland State University School of Public Health, Portland, OR USA; 2 Harvard University, Harvard Kennedy School, Cambridge, MA USA

## Abstract

**Background:**

Service-sector workers in the U.S. face extremely limited access to paid family and medical leave, but little research has examined the consequences for worker wellbeing. Our objective was to determine whether paid leave was associated with improved economic security and wellbeing for workers who needed leave for their own serious health condition or to care for a seriously ill loved one.

**Methods:**

We analyzed data collected in 2020 by the Shift Project from 11,689 hourly service-sector workers across the US. We estimated the impact of taking paid leave on economic insecurity and wellbeing relative to taking unpaid leave, no leave, or not experiencing a need to take leave.

**Results:**

Twenty percent of workers needed medical or caregiving leave in the reference period. Workers who took paid leave reported significantly less difficulty making ends meet, less hunger and utility payment hardship, and better sleep quality than those who had similar serious health or caregiving needs but did not take paid leave.

**Conclusions:**

Access to paid leave enables front line workers to take needed leave from work while maintaining their financial security and wellbeing.

**Supplementary Information:**

The online version contains supplementary material available at 10.1186/s12889-021-11999-9.

## Background

Workers experience a range of issues that may necessitate leave from work, including for their own medical needs and for caregiving, but the extent to which paid leave policies support their economic security and wellbeing during these times remains unknown. In contrast to other high-income countries, the US does not mandate any paid leave for one’s own short- or long-term illness (usually referred to as sick leave and medical leave, respectively), caring for sick family members (family caregiving leave), or bonding with a new child (parental leave, usually covered under family/medical leave policies) [1–4]. Instead, workers rely on a patchwork of state and local paid sick and paid family and medical leave policies, or on their employers’ voluntary benefits. Public policies are expanding but remain limited: by mid-2021, just 11 states and 30 cities had passed paid sick leave policies, and nine states and the District of Columbia had passed paid family and medical leave (PFML) laws [5]. This translates to only 75% of private sector workers with access to paid sick leave and 20% with access to paid family leave [6]. Paid sick and family leave are even less accessible among workers in service, retail, and construction jobs and those in part-time and low-wage jobs [6].

The health and economic effects of access to paid *sick* leave (usually for short-term illness) and paid *parental* leave are relatively well known. For example, workers with access to paid sick leave benefits are less likely to report to work while sick [7], to forgo medical care for themselves and their family members [8], and to experience workplace injury [9]. They are more likely to experience improved sleep quality [10] and to seek cancer screening and other preventive medical care [11]. Children whose parents have access to paid sick leave have improved access to and use of healthcare services and reduced ER visits [12]. Paid parental leave for new parents has been linked to improved self-rated health [13], reduced psychological distress [13, 14], and reduced alcohol consumption [13]. Benefits of paid leave further spillover to children through increased breastfeeding [15, 16] and reduced late vaccinations [17].

Much less is known, however, about how paid family and medical leave policies support workers taking leave for their own serious medical condition or to care for a seriously ill family member, in particular, when the need for leave lasts more than a few days. This is despite the fact that more than half of all Family and Medical Leave Act (FMLA) claims were for medical leave [18] and 60% of the 53 million family caregivers in the U.S. are employed [19].

Access to PFML has been shown to act as a buffer in the relationship between caring for family members with special health needs and poor mental health [20] and appears to help middle-aged female caregivers remain in the workforce [21]. In a context where 41% of US households report not having enough savings to cover a $2000 financial shock [22], the lack of PFML could be financially devastating for workers attempting to follow public health guidance to stay home when sick.

PFML may impact family economic security and worker wellbeing through multiple pathways. In their study of employed patients undergoing bone marrow transplantation to treat advanced blood cancer, Abelda and colleagues examine the impact of access to paid leave on patient-reported health measures both directly and indirectly through financial burden (i.e., lack of satisfaction with family finances, difficulty with monthly payments, and not having enough money at the end of the month) [23]. Importantly, they find evidence for both pathways and that health was improved when the patient and, independently, their caregiver had access to paid leave, reinforcing the need for both medical and caregiving leave.

These few studies directly examining paid medical and caregiving leave begin to illuminate potential health and economic impacts, but none has yet measured the prevalence of needed medical or caregiving leave, differentiated unpaid from paid leave, or focused on a broad set of economically vulnerable workers. The purpose of our study is to determine whether workers who experienced a need for medical or caregiving leave and who took paid leave had improved economic security and wellbeing relative to similar workers who took no leave or only unpaid leave. Because the literature on medical and caregiving leave, in contrast to parental leave, is so sparse, we focus on workers who needed leave for these reasons. We focus on service and retail workers—a group that has found themselves on the front lines of the COVID-19 pandemic response and bearing a significant risk of exposure to infection.

## Data and methods

### Data

We draw on unique data collected by the Shift Project, of which Dr. Schneider is the Co-PI. The Shift Project is an ongoing repeated cross-sectional survey that, beginning in 2017, collects two new cross-sections each year. The Shift Project uses Facebook/ Instagram as both quasi-sampling framing and a recruitment device, using Facebook’s sophisticated ad targeting system to construct “audiences” of workers at specific large named service-sector firms. The Shift Project then recruits these workers to the online survey by fielding paid advertisements that appear in workers’ newsfeeds on desktop and mobile. This approach is low-cost, very flexible, and allows for rapid-response data collection.

In this instance, the Shift Project approach allowed for the authors to undertake mid-COVID-19-pandemic collection of detailed data on qualifying events and leave-taking alongside economic outcomes for a large sample of vulnerable workers. To do so, we designed a new survey module on qualifying events and paid leave that we added to the Shift Project survey and that was fielded to 11,689 hourly service-sector workers at 119 large firms (a full list of these firms is provided in Additional file 1: Appendix Table A1) in two repeated cross-sections. A first group of respondents was surveyed between March and May of 2020 and a second independent group was surveyed between September and November of 2020.

Existing data on paid family and medical leave are much more limited than the data we draw on here. Few data sources, with the important exception of the ATUS Leave Module fielded in 2011 and 2017–2018, distinguish paid from unpaid leave, examine leave for reasons other than childbirth or bonding with a new child, or identify the source of pay (e.g., employer versus through a government program). The Shift Project data are the only large-scale data source that asks these detailed questions along with information on household economic security and worker subjective wellbeing.

However, these data are drawn using a non-probability sampling approach and Facebook-based survey data collection has a low response rate [24]. In this instance 12.2% of Facebook/ Instagram users who saw the ad, clicked-through to the survey and 10.4% of those who clicked-through contributed survey data, such that 1.3% of those to whom the ad was displayed contributed survey data. While this response rate is low, prior methodological work finds that univariate distributions and multi-variate associations in Shift replicate those in “gold-standard” data sources such as the NLSY and Current Population Survey [24] and these data have been used to examine the correlates and consequences of precarious job quality [7, 25, 26]. In these analyses, we weight the Shift Project sample to the characteristics of workers in the same occupations and industries in the American Community Survey on race/ethnicity, gender, and age and employ these weights in all of our estimates. Additional file 1: Appendix Table A2 contrasts the demographics of respondents in service-sector occupations with those of the weighted and unweighted analysis sample from the Shift Project data.

### Measures

#### Leave taking

All survey respondents are asked if they experienced any of three types of events that would “qualify” them for paid leave under most existing state laws and company policies. Respondents were asked if they (1) “welcomed a new child into their family through birth, adoption, or foster placement” (not included in this analysis), (2) “had a serious health condition or illness, like recovering from a surgery or serious illness,” and (3) “have needed to care for seriously ill or injured family member.” For each item, respondents interviewed between March and May were asked about their experience with each event since January 1 of 2020; respondents interviewed between September and November of 2020 were asked about the prior 12 months. In this analysis, we focus on medical and caregiving events, dropping 368 respondents who reported a new child as their only qualifying event. At both waves, respondents could report more than one type of event, though respondents who experienced multiple events are asked about leave-taking for the combined event (i.e., “Did you take leave from your job to care for yourself or others?”). Reporting retrospectively on a reference period with a mean length of 7 months, 20% of these workers had experienced a need for medical or caregiving leave.

Respondents who reported at least one qualifying event were asked if they took leave from their job in response and, if they did, if they received pay from their employer, with options of receiving full pay, partial pay, or no pay. We draw on data from these two sets of measures to construct our key independent variable. We code respondents into four mutually exclusive categories: (1) did not experience any qualifying event, (2) experienced a qualifying event, but did not take leave, (3) experienced a qualifying event, and took unpaid leave, and (4) experienced a qualifying event, and took paid leave.

#### Economic insecurity

We construct four indicators of household economic insecurity. First, we measure if respondents currently find it “very difficult” (1) to cover expenses and pay bills versus finding it “somewhat” or “not at all” difficult (0). Second, we gauge if respondents reported that they could probably or certainly not come up with $400 in response to an unexpected need within the next month versus being probably able or certainly able to do so (0). Third, we distinguish respondents who did not pay the full amount of a gas, oil, or electric bill in the past month (1) from those who paid these bills in full (0). Finally, we measure hunger hardship if respondents report either receiving free food or meals because they didn’t have enough money or going hungry but not eating because they couldn’t afford enough food in the last month.

#### Wellbeing

In addition to these measures of economic security, we also measure two indicators of wellbeing. Respondents are asked “in general, how would you say things are these days?” We distinguish respondents who report being “very happy” or “pretty happy” (1) from those who are “not too happy” (0). We also measure respondents’ reported sleep quality during the past month, distinguishing those with “very good” or “good” sleep (1) from those with “fair” or “poor” sleep (0).

#### Controls

We measure and control for a set of demographic characteristics: gender; race/ethnicity (white, non-Hispanic; Black, non-Hispanic; Hispanic; other or multiple race/ethnicities, non-Hispanic); marital status (single, cohabiting, married); age; having children ages 0 to 4, ages 5 to 9, ages 10 to 14, and ages 15 to 18; current school enrollment; and educational attainment (< HS; HS/GED; some college; Associates degree; Bachelors degree; Masters degree or more). We also measure and control for a set of job characteristics: job tenure, union coverage, hourly wage, and number of usual work hours. Finally, we include a set of month and state fixed-effects. In a supplementary set of models, we also introduce controls for type of qualifying event (own health versus caregiving) and then for self-rated health.

### Models/approach

To estimate the consequences of not taking paid leave when needed, we estimate ordinary least squares (OLS) regression models of leave-taking on a set of outcome measures that capture household economic insecurity and worker wellbeing.

Our analytic approach relies on exploiting the implicit time-ordering of events in these cross-sections. The leave-taking module asks about events in the recent past and we then gauge outcomes measured at the time of survey or in reference to the month prior. This survey structure allows us to correctly time-order qualifying events, leave-taking, and outcomes. The controls are measured at the time of survey.

Additionally, we note that recruiting workers employed at specific firms imposes a significant scope condition on the data. All respondents, in both Spring 2020 and Fall 2020, were employed at the time of survey. This means that our analyses only pertain to workers who returned to employment following a qualifying event. Workers who stopped working following a qualifying event are not included in the data.

These estimates are threatened by several potentially confounding processes. One possibility is that respondents who take paid leave may face the most severe health challenges or intensive caregiving responsibilities. That is, there may be negative selection into leave-taking. It is also possible that respondents who are able to take paid leave may be positively selected in being more knowledgeable about company policies or better able to navigate state systems. While the direction of bias is unclear, these models risk mistaking a spurious association between taking paid leave and worker outcomes for a causal one. In addition to the set of controls for possible confounders described above, we guard against these risks of confounding in several ways.

First, to guard against the risk of negative selection into leave-taking, we compare workers who took paid leave to those who took unpaid leave. We expect that workers who took paid leave will fare significantly better than both those who took unpaid leave and those with qualifying events who took no leave. However, we expect that workers without any qualifying events will fare best.

Second, as a robustness test, focusing only on workers with qualifying events, we control for the type of qualifying event, distinguishing medical and caregiving leave, and we then separately introduce a control for self-rated health as a conservative proxy for severity of event. While this risks “over-controlling” (in so far as not taking leave may make for worse health), it is also a powerful safeguard against negative selection into paid leave-taking.

Finally, to examine the risk of bias from different recall periods (i.e., two months for those interviewed in March 2020 to 12 months for those interviewed between September and November 2020), we conducted two additional sensitivity tests. We first control for implied maximum duration of the recall period. We then interact the maximum duration of recall period with our key independent variable and re-estimate all of the regression models.

## Results

Ten percent of our sample experienced a need for caregiving leave and 11% experienced a need for medical leave in the reference period (Table 1). Our weighted sample was nearly evenly split between women (52%) and men (48%). The sample was 63% non-Hispanic white, 9% non-Hispanic Black, and 20% Hispanic, with the remaining 8% identifying with another or multiple race/ethnicities, with a mean age of 38. Fifty-two percent were single, 29% married, and 19% cohabiting. Eight percent of respondents reported a child under the age of 5, 9% a child aged 5 to 9, 11% a child aged 10 to 14, and 12% reported having a child aged 15 to 18. A little more than a fifth of respondents were enrolled in school. Only 14% had a Bachelors degree. A majority had been at their job for at least 3 years, with one-third reporting 6 years or more, but 19% had less than 1 year of job tenure. On average, respondents in our sample worked 34 h per week and earned $14/h.

**Table 1:** Descriptive Statistics, Weighted

*Leave Events*	
Care Event	10%
Health Event	11%
*Leave Taking*	
Event, No Leave	9%
Event, Unpaid Leave	5%
Event, Paid Leave	4%
No Event	81%
*Gender*	
Female	52%
Male	48%
*Race/Ethnicity*	
White, non-Hispanic	63%
Black, non-Hispanic	9%
Hispanic	20%
Other, non-Hispanic	8%
*Marital Status*	
Single	52%
Cohabiting	19%
Married	29%
*Children*	
Age 0 to 4	8%
Age 5 to 9	9%
Age 10 to 14	11%
Age 15 to 18	12%
*Enrolled in School*	21%
*Age (mean)*	38
*Educational Attainment*	
Less than HS	4%
HS/GED	32%
Some College	38%
Associates Degree	12%
Bachelors Degree	12%
Masters Degree or more	2%
*Job Tenure*	
Less than 1 year	19%
1 year	13%
2 years	13%
3 years	10%
4 years	8%
5 years	7%
6 years or more	31%
*Union*	11%
*Usual Weekly Work Hours (mean)*	34
*Hourly Wage (mean)*	$14
N	11,689

Workers with a qualifying event who took no leave or unpaid leave reported difficulty making ends meet, inability to cope with a $400 shock, and recent hunger and utility hardship at higher rates than both workers who experienced a qualifying event and took paid leave and those who did not experience a qualifying event (Table 2). Additionally, workers with a qualifying event who took no leave or unpaid leave reported lower levels of happiness and sleep quality than workers who took paid leave or who did not have a need for leave.

**Table 2:** Bivariate Association between Leave-Taking and Economic Security and Wellbeing

	Event, No Leave	Event, Unpaid Leave	Event, Paid Leave	No Event	All	x^2^
Difficulty Making Ends Meet	27%	28%	17%	14%	16%	*p* < .001
Cannot Cope with $400 Shock	46%	44%	33%	31%	33%	*p* < .001
Hunger Hardship Last Month	27%	26%	13%	14%	15%	*p* < .001
Utility Hardship Last Month	27%	25%	16%	14%	15%	*p* < .001
Very/Pretty Happy	62%	63%	76%	73%	72%	*p* < .001
V. Good/Good Sleep Quality	23%	24%	36%	37%	35%	*p* < .001
N	1087	704	459	9439	11,689	

Compared to workers with a qualifying event who took unpaid leave, those who took paid leave have significantly lower economic insecurity and higher wellbeing (Table 3). After controlling for confounders, workers who took paid leave were 8 percentage points less likely to report difficulty making ends meet (*p* < 0.05), 9 points less likely to have experienced hunger hardship (*p* < 0.01) in the prior month, and 8 points less likely to have experienced utility payment hardship in the prior month (*p* < 0.05). Respondents who took paid leave for a qualifying event were also 11 percentage points more likely to report being very/pretty happy (*p* < 0.01) and 10 percentage points more likely to report very good/good sleep quality (*p* < 0.01) than those who took unpaid leave. Workers with a qualifying event who did not take any leave reported economic insecurity and wellbeing at similar levels as those who took unpaid leave only. Workers who did not experience a need for any leave had the lowest levels of economic insecurity and highest wellbeing. See Additional file 1: Appendix Table A3 for full model specification.

**Table 3:** Association between Paid Leave Taking and Economic Security/Wellbeing

	(1)	(2)	(3)	(4)	(5)	(6)
	Difficulty Making	Cannot Cope with	Hunger Hardship	Utility Hardship	Very/Pretty Happy	V. Good/Good Sleep
	Ends Meet	$400 Expense	Last Month	Last Month		
*Type of Leave*						
Event, No Leave	−0.02	0.02	0.01	0.01	0.00	0.01
	(0.03)	(0.03)	(0.03)	(0.03)	(0.03)	(0.03)
Event, Unpaid Leave	Ref.	Ref.	Ref.	Ref.	Ref.	Ref.
Event, Paid Leave	−0.07*	−0.03	−0.08**	− 0.07*	0.11**	0.10**
	(0.03)	(0.04)	(0.03)	(0.03)	(0.03)	(0.04)
No Event	−0.13***	−0.10***	− 0.10***	− 0.09***	0.11***	0.14***
	(0.02)	(0.02)	(0.02)	(0.02)	(0.02)	(0.02)
Observations	11,689	11,689	11,689	11,689	11,689	11,689

*Note*: Estimates from linear probability models that include controls for gender, race/ethnicity, age, marital status, having children age 0 to 4, age 5 to 9, age 10 to 14, age 15 to 18, school enrollment, educational attainment, job tenure, union membership, usual work hours, and hourly wage, as well as month and state fixed-effects. ∗∗∗ = *p* < .001; ∗∗ = *p* < .01; ∗ = *p* < .05

Additional file 1: Appendix Fig. A1 shows the fairly sharp separation between the economic security and wellbeing of workers who experienced a qualifying event and took no leave or unpaid leave versus those who did not experience a qualifying event or had an event but took paid leave. Despite experiencing a significant health or caregiving challenge, those with paid leave consistently fared quite similarly to those who did not experience the event on these outcomes.

To determine whether our results reflect negative selection into leave-taking, we first control for the type of qualifying event (caregiving vs. medical need) and we find no notable attenuation in the key associations (Additional file 1: Appendix Table A4). We also introduce a control for self-rated health in order to guard against the risk that severity of illness might drive unpaid leave taking in particular and thus bias our estimates. Here too, we continue to find consistent evidence that workers who experienced qualifying events and were able to take leave fared significantly better than those who took unpaid leave. Sensitivity analyses to examine differential recall did not change our results.

## Discussion

We find evidence that paid leave is associated with reduced economic insecurity and improved wellbeing for workers facing serious medical conditions, whether for themselves or their family members. Workers who are able to take paid leave when experiencing a qualifying event report significantly less difficulty making ends meet, less hunger and utility payment hardship, and better sleep quality than those who had similar serious health or caregiving needs but did not take paid leave. Indeed, we did not observe such an association for unpaid leave: workers with a qualifying event who took unpaid leave reported similar levels of economic insecurity and wellbeing as workers who took no leave, reinforcing the critical role of pay during leave. This is consistent with prior research that shows significant health impacts of paid, but not unpaid, leave [27].

It is not clear why workers with a qualifying event who did not take any leave experienced worse economic hardship than workers who took paid leave. Workers who did not take leave would have continued in paid employment, while some workers who took paid leave would have received only partial pay. One possibility is that, rather than take leave, some workers reduced their hours. Another possibility is that these workers had to hire paid caregivers to meet their needs, thus imposing an additional economic burden. Future research should further explore these mechanisms.

As hypothesized, workers who experienced a qualifying event during the study period, regardless of their leave-taking status, fared worse than workers who did not experience such an event. This suggests that access to paid leave alone does not erase the challenges that come with many of the reasons for needing leave. One might expect that happiness and sleep quality would be affected by the underlying medical condition necessitating leave, or the stress associated with caring for a sick family member; however, policy choices can ensure that financial insecurity does not necessarily follow. A comprehensive safety net, including fully paid, job-protected leave and other supports like universal health insurance, could alleviate the financial risks associated with experiencing a health event, particularly for the two in five US households reporting not enough savings to cover a $2000 financial shock [22]. This concern is heightened for people who face multiple serious health or care events within a brief time-period. These workers may exhaust any available paid leave and so face heightened consequences of the second event, reflecting the limits of existing paid leave programs.

The Families First Coronavirus Response Act (FFCRA) provided temporary relief for some workers during COVID who needed to stay home while sick or in quarantine or to care for a family member who was sick, quarantined, or without school. However, because the FFCRA excluded large (as well as very small) firms, most workers in our sample did not benefit from these provisions. This mirrors other paid leave laws that exclude small firms and/or certain types of workers, disproportionately impacting economically vulnerable workers. A recent study of San Francisco’s Paid Parental Leave Ordinance found that, by limiting to firms with at least 20 workers, low-income workers were disproportionately excluded [28].

While the evidence base documenting the importance of paid leave to support new parents has grown more robust, much less research has examined workers who need leave for other purposes. By focusing on medical and caregiving leave, we expand the literature on the health and economic benefits of paid family and medical leave to cover a wider set of workers. Furthermore, we focus on service and retail workers – a diverse set of workers whose needs for and access to leave have been understudied, particularly in light of their critical role on the front lines of the COVID-19 pandemic response.

Our sample includes only workers who were employed at the time they completed the survey. Thus, our analyses pertain only to workers who returned to employment following a qualifying event, while workers who stopped working following a qualifying event are not included in the data. Our results may underestimate the link between paid leave and financial security and improved wellbeing if workers without paid leave were more likely to quit their jobs when facing a qualifying event. Some of the respondents in our sample who reported paid leave may have received only partial pay, but our sample size did not allow us to disaggregate fully- and partially-paid leave. We may therefore be underestimating the impact of access to fully-paid leave on financial insecurity and wellbeing.

We are confident that our survey allows for the correct time-ordering of qualifying events, leave-taking, and outcomes. However, there is a possibility, though unlikely, that a very rapid series of events could have unfolded that would lead to bias in our results. For example, bias might occur if, within a period of thirty days, a respondent first experienced hunger or utility hardship or diminished happiness or sleep quality which then *caused* a serious medical issue or a caregiving issue, in response to which the respondent then needed to either take leave or not take leave. While possible, this sequence of events, with this pattern of causality, and on this compressed time frame, in our judgement, is unlikely to present a risk of serious bias. Another potential source of bias is differential recall. We minimize this threat by focusing on major life events that are unlikely to be forgotten within a year and by asking clear, directed questions about exposures. Sensitivity analyses to examine differential recall by the implied recall window did not effect our results. Finally, we cannot rule out the possibility that unobserved characteristics result in a spurious correlation between paid leave-taking and our outcomes. We attempt to minimize this concern by controlling for a robust set of potential confounders and by comparing workers who took paid leave to those who took unpaid leave (rather than using those who did not take any leave as our primary control group); however, we cannot confidently claim a causal relationship.

While the Shift Project data include more details about paid family and medical leave availability and leave-taking than most other surveys, there remain gaps in what we can explain with our data. We encourage future studies to include even more detailed questions that would enable a deeper understanding of leave-taking behavior, including differentiating respondents with qualifying events who did not take leave because they did not have any paid leave, did not want to use their available leave, or had already used all of their employer-provided leave.

## Conclusions

Lack of access to paid leave has consequences for workers, their families, and population health. The COVID-19 pandemic has underscored the critical public health importance of access to paid family and medical leave. Expanding paid family and medical leave policies would enable front line workers to take needed leave from work while maintaining their financial security and wellbeing.

## Supplementary Information

**Additional file 1.**

## Data Availability

The datasets generated and/or analysed during the current study are not publicly available due to the need to preserve respondent confidentiality but are available from the corresponding author on reasonable request.

## Abbreviations

FMLA: Family and Medical Leave Act
GED: General Educational Development
HS: High School
NLSY: National Longitudinal Survey of Youth
OLS: Ordinary Least Squares
PFML: Paid Family and Medical Leave
US: United States
