# Supplementary material for: The association of paid medical and caregiving leave with the economic security and wellbeing of service sector workers
Source: BMC Public Health. 2021 Nov 1;21:1969. doi: 10.1186/s12889-021-11999-9 (PMC8558760; doi:10.1186/s12889-021-11999-9)
Supplement: Supplementary file 1 — Additional file 1. [file 12889_2021_11999_MOESM1_ESM.docx]

APPENDIX TABLES

**Table A1. Firms at Which Respondents were Employed**

**Table A2. Comparison of Workers in American Community Survey (Weighted, 2018 and 2019) and Shift Project Data (Weighted, 2020)**

|  | ACS | Shift |
| --- | --- | --- |
| *Gender* |  |  |
| Male | 47% | 48% |
| Female | 53% | 52% |
|  |  |  |
| *Race/Ethnicity* |  |  |
| White, non-Hispanic | 54% | 63% |
| Black, non-Hispanic | 13% | 9% |
| Hispanic | 23% | 19% |
| Other/Two or more races, non-Hispanic | 10% | 8% |
|  |  |  |
| *Education* |  |  |
| *High School or Less* | 50% | 35% |
| *Some College* | 30% | 38% |
| *Associate’s Degree* | 8% | 12% |
| *Bachelor’s Degree or more* | 12% | 14% |
|  |  |  |
| *Enrolled in School* | 21% | 20% |
|  |  |  |
| *Age* |  |  |
| Mean | 36 | 38 |
| Median | 29 | 35 |
|  |  |  |
| N | 296,645 | 11,781 |

**Table A3. Full Model Results**

Cont….

**Table A4. Association Between Paid Leave Taking and Economic Security/Wellbeing, Robustness (Conditional on Qualifying Event)**

|  | (1) | (2) | (3) | (4) | (5) | (6) | |
| --- | --- | --- | --- | --- | --- | --- | --- |
|  | Difficulty Making | Cannot Cope with | Hunger Hardship | Utility Hardship | Very/Pretty | V. Good/Good |  |
|  | Ends Meet | $400 Expense | Last Month | Last Month | Happy | Sleep | |
| *Baseline Controls*  Event, No Leave | -0.01 | 0.04 | 0.02 | 0.01 | -0.01 | 0.01 | |
| Event, Unpaid Leave | (ref) | (ref) | (ref) | (ref) | (ref) | (ref) | |
| Event, Paid Leave | -0.08* | -0.03 | -0.07* | -0.09** | 0.10** | 0.10** | |
| *+ Control for Type of Event*  Event, No Leave | -0.01 | 0.03 | 0.03 | 0.02 | -0.01 | 0.00 | |
| Event, Unpaid Leave | (ref) | (ref) | (ref) | (ref) | (ref) | (ref) | |
| Event, Paid Leave | -0.08* | -0.03 | -0.08** | -0.09** | 0.11** | 0.11** | |
| *+ Control for Self-Rated Health*  Event, No Leave | -0.01 | 0.02 | 0.03 | 0.02 | -0.01 | 0.00 | |
| Event, Unpaid Leave | (ref) | (ref) | (ref) | (ref) | (ref) | (ref) | |
| Event, Paid Leave | -0.07* | -0.02 | -0.06* | -0.08* | 0.09** | 0.08* | |
| Observations | 2,250 | 2,250 | 2,250 | 2,250 | 2,250 | 2,250 | |

*Note*: Estimates from linear probability models that include controls for gender, race/ethnicity, age, marital status, having children age 0 to 4, age 5 to 9, age 10 to 14, age 15 to 18, school enrollment, educational attainment, job tenure, union membership, usual work hours, and hourly wage, as well as month and state fixed-effects. ∗∗∗ = *p* < .001; ∗∗ = *p* < .01; ∗ = *p* < .05

**Figure A1: Predicted Values of Outcomes by Paid Leave Status**

*Note*: Estimates from models following same specification as Table 3.
